# Predicting Body Mass Index From Structural MRI Brain Images Using a Deep Convolutional Neural Network

**DOI:** 10.3389/fninf.2020.00010

**Published:** 2020-03-20

**Authors:** Pál Vakli, Regina J. Deák-Meszlényi, Tibor Auer, Zoltán Vidnyánszky

**Affiliations:** ^1^Brain Imaging Centre, Research Centre for Natural Sciences, Budapest, Hungary; ^2^School of Psychology, Faculty of Health and Medical Sciences, University of Surrey, Guildford, United Kingdom

**Keywords:** deep learning, convolutional neural networks, magnetic resonance imaging, body mass index, caudate nucleus, amygdala

## Abstract

In recent years, deep learning (DL) has become more widespread in the fields of cognitive and clinical neuroimaging. Using deep neural network models to process neuroimaging data is an efficient method to classify brain disorders and identify individuals who are at increased risk of age-related cognitive decline and neurodegenerative disease. Here we investigated, for the first time, whether structural brain imaging and DL can be used for predicting a physical trait that is of significant clinical relevance—the body mass index (BMI) of the individual. We show that individual BMI can be accurately predicted using a deep convolutional neural network (CNN) and a single structural magnetic resonance imaging (MRI) brain scan along with information about age and sex. Localization maps computed for the CNN highlighted several brain structures that strongly contributed to BMI prediction, including the caudate nucleus and the amygdala. Comparison to the results obtained via a standard automatic brain segmentation method revealed that the CNN-based visualization approach yielded complementary evidence regarding the relationship between brain structure and BMI. Taken together, our results imply that predicting BMI from structural brain scans using DL represents a promising approach to investigate the relationship between brain morphological variability and individual differences in body weight and provide a new scope for future investigations regarding the potential clinical utility of brain-predicted BMI.

## Introduction

Over the last few years, the use of deep learning (DL) has become increasingly widespread in the analysis of neuroimaging data in several different application domains (Arbabshirani et al., 2017; Litjens et al., 2017; Shen et al., 2017; Zaharchuk et al., 2018; Davatzikos, 2019). DL is a branch of machine learning that allows the construction of computational models that learn to represent data at increasing levels of abstraction to solve specific tasks (LeCun et al., 2015; Goodfellow et al., 2016). Among DL methods, deep convolutional neural networks (CNNs) (LeCun et al., 1990; Lecun et al., 1998), which are widely adopted in the computer vision community due to their capability to achieve outstanding object detection performance (Krizhevsky et al., 2012), represent a promising approach to analyzing brain imaging data in studies of psychiatric and neurological disorders (Vieira et al., 2017; Durstewitz et al., 2019). The majority of studies employing CNNs used structurl and/or functional magnetic resonance imaging (MRI) data to examine patients with Alzheimer’s disease and mild cognitive impairment (Gupta et al., 2013; Payan and Montana, 2015; Sarraf and Tofighi, 2016; Farooq et al., 2017; Meszlényi et al., 2017; Hosseini-Asl et al., 2018; Islam and Zhang, 2018; Basaia et al., 2019); although there are examples of studies classifying other mental disorders as well, such as attention-deficit hyperactivity disorder (Zou et al., 2017) and alcoholism (Wang et al., 2017).

The potential of these methods lies partly in that—in contrast to conventional mass univariate analytical methods—machine learning in general and DL in particular allow statistical inferences at the individual level (Vieira et al., 2017). Besides the diagnosis of brain disorders, machine learning can also be used to identify individual differences in the brain aging process (Cole and Franke, 2017; Cole et al., 2019). DL methods are increasingly prevalent in this application area as well, as CNNs can be used to predict the chronological age of individual subjects based on structural brain MRI scans with a mean absolute error (MAE) of 4.16 years (Cole et al., 2017). Comparable results can be obtained with CNNs using whole-brain functional connectivity patterns, derived from resting-state fMRI data, as input (Li et al., 2018; Vakli et al., 2018). These findings bear significance for two main reasons. First, they provide proof of concept that a single MRI scan contains information that is strongly related to chronological age (Cole and Franke, 2017). Second, they provide a means to quantify the individual risk of age-related cognitive decline and disease. In fact, several studies have shown that an increase in brain-predicted age relative to chronological age is associated with various neurological and psychiatric disorders, poorer physical fitness, and increased risk of mortality (Franke and Gaser, 2012; Koutsouleris et al., 2014; Cole et al., 2015, 2018; Habes et al., 2016; Löwe et al., 2016; Pardoe et al., 2017).

The above findings demonstrate how computational models aimed at predicting a certain biometric trait have potential clinical applicability. Here we investigated whether structural brain imaging and machine learning can be used for predicting a physical trait that is of significant clinical relevance—the body mass index (BMI) of the individual. The prevalence and disease burden of excessive body weight is on the rise globally (The GBD 2015 Obesity Collaborators, 2017), and there is extensive evidence showing a relationship between obesity—defined as a BMI greater than 30 kg/m^2^—and brain health. In particular, a number of studies have shown that obesity and associated cardiovascular disease and metabolic disorders in midlife are related to cognitive impairment and dementia in later life (Pedditizi et al., 2016; Dye et al., 2017; Alford et al., 2018; Singh-Manoux et al., 2018). To date, a large number of studies using conventional neuroimaging methods have investigated the differences in brain structure and function between obese/overweight and lean individuals. Increased BMI has been associated with reduced gray matter volume (Pannacciulli et al., 2006; Taki et al., 2008; Raji et al., 2010; Brooks et al., 2013) and white matter integrity (Stanek et al., 2011; Kullmann et al., 2015). Altered resting-state functional connectivity (Avery et al., 2017) and activation to visual food cues in brain regions involved in reward processing and inhibitory control (Carnell et al., 2012; Pursey et al., 2014; Val-Laillet et al., 2015) have also been described in obese individuals. A recent study has investigated the associations between obesity, regional gray matter volumes, and white matter microstructure, as assessed by MRI, in a large sample of 12,087 participants (Dekkers et al., 2019). The authors have found sex differences in the relationship between total body fat percentage and the volume of several subcortical regions of the brain reward system, and contrary to previous findings, a positive association between total body fat percentage and white matter microstructural coherence.

Training a machine learning algorithm to predict individual BMI based on brain imaging data has several potential applications. On the one hand, once sufficiently accurate prediction performance is achieved, it is possible to investigate which features (e.g., structural properties of the brain) contribute significantly to the predicted value. This has the potential to provide complementary information regarding the relationship between brain structure and body weight, besides conventional neuroimaging approaches. On the other hand, it can pave the way for potential clinical applications, inasmuch as the discrepancy between the true and the predicted BMI might be related to individual differences in food intake regulation and associated propensity for future weight gain. This would be analogous to that how the difference between brain-predicted and chronological age is used to quantify health risks.

Here we apply, for the first time to our knowledge, DL to predict individual BMI based on brain imaging data. In particular, we employ a CNN for BMI prediction based on T1-weighted structural MR images, as well as information about the participants’ age and sex. This approach has the advantage of being able to use minimally preprocessed neuroimaging data as input and automatically learn a hierarchical set of representations suitable for solving the task at hand (LeCun et al., 2015), as opposed to conventional neuroimaging and machine learning methods that rely on *a priori* manual extraction of features from raw data (Vieira et al., 2017). Based on the findings discussed above, we hypothesized that BMI could be accurately predicted based on a single MRI bran scan, and hence a CNN can be trained to effectively perform this task on novel scans as well.

Once a well-performing model has been obtained and tested on new data, a logical next step is to try to make sense of why the model predicts what it predicts. While deep neural networks are usually regarded as “black boxes,” it is possible to give reasonable explanations for their predictions without elucidating the underlying mechanisms (Lipton, 2016). Common approaches include projecting hidden layer activations back to input space to find patterns that excite feature maps the most (Zeiler and Fergus, 2014), examining the effect of occluding different parts of the input image on model performance (e.g., Vakli et al., 2018), or identifying those pixels in the input image that have the greatest impact on the model’s predictions (e.g., Simonyan et al., 2013). With regard to the latter approach, a particular method that has been used extensively in recent years to provide “visual explanations” for CNNs’ decisions is Gradient-weighted Class Activation Mapping (Grad-CAM) (Selvaraju et al., 2017). This technique uses the gradient information flowing into the last convolutional layer of the CNN to highlight image regions that played an important role in predicting a certain target concept. Here we adapted this method to the context of regression based on 3D images to localize brain regions that made a significant contribution to BMI prediction.

Since the present study represents one of the first attempts to apply Grad-CAM for analyzing neuroimaging data, we also intended to investigate the neural underpinnings of individual differences in body weight using a more conventional neuroimaging approach and compare the obtained results. To this end, we performed automatic anatomical processing using the FreeSurfer software and general linear modeling to examine the relationship between brain morphology and BMI. FreeSurfer implements the automatic reconstruction of the cortical surface as well as subcortical structure segmentation using a probabilistic atlas (Dale et al., 1999; Fischl et al., 1999). The simultaneous application of the DL and automatic segmentation methods was motivated by the possibility that, as compared to this more conventional latter approach, using minimally preprocessed anatomical images and representation learning paired with gradient-based visualization would yield complementary evidence regarding the relationship between brain structure and body weight.

## Materials and Methods

### Dataset

All analyses reported in this article include participants from the UK Biobank population cohort^1^. UK Biobank is a large prospective study comprising around 500,000 individuals recruited between 2006 and 2010 from across Great Britain who underwent physical and cognitive assessment, provided biological samples and completed questionnaires examining health and lifestyle (Allen et al., 2012). A subset of the participants (*N* = 22,392) underwent additional MRI from May 2014 until the data release in October 2018. Participants with a self-reported history of cancer, stroke, heart attack, deep-vein thrombosis, or pulmonary embolism diagnosed by a medical doctor (based on data-fields 2453, 6150, and 6152) were omitted from the current study. Additionally, only participants whose body mass indices were reported at the time of the imaging visit (data-field 21,001 instance 2) were included in the analyses. Finally, participants with a raw T1-weighted structural image deemed “unusable” by the UK Biobank team were also excluded. Image quality control on behalf of UK Biobank consisted of the rough manual review of T1 images supplemented by a beta-version automated quality control pipeline (Alfaro-Almagro et al., 2018). Eventually, 9518 females, aged between 45 and 80 years (mean ± SD = 62.11 ± 7.30 years), and 8420 males, aged between 44 and 80 years (mean ± SD = 63.21 ± 7.59 years), were included in the present study. For females, BMI ranged between 13.39 and 58.70 kg/m^2^ (mean ± SD = 26.15 ± 4.72 kg/m^2^), while for males, it ranged between 16.67 and 58.04 kg/m^2^ (mean ± SD = 27.03 ± 3.99 kg/m^2^).

All participants provided informed consent to participate in the UK Biobank study. The UK Biobank Research Ethics Committee (REC) approval number is 11/NW/0382. Detailed information on the consent procedure of UK Biobank are available at the following URL: http://biobank.ctsu.ox.ac.uk/crystal/field.cgi?id=200.

### Data Acquisition and Preprocessing

#### Neuroimaging

Data were acquired on Siemens Skyra 3T MRI scanners (Siemens Healthcare, Erlangen, Germany) at the UK Biobank imaging centers in Cheadle, Newcastle, and Reading. A standard Siemens 32-channel RF receive head coil was applied. The brain imaging protocol included a T1-weighted 3D magnetization-prepared rapid gradient echo (MPRAGE) sequence for structural imaging, using in-plane acceleration (iPAT = 2) and a field-of-view (FOV) of 208 × 256 × 256 with isotropic 1 mm spatial resolution.

Raw T1-weighted images were preprocessed by the UK Biobank team using an automated processing pipeline based on FSL tools (Jenkinson et al., 2012). The preprocessing pipeline included gradient distortion correction, cutting down the FOV, skull stripping, and non-linear transformation to MNI152 space (Alfaro-Almagro et al., 2018). In-house preprocessing was limited to reducing the size of the images to ease the computational burden of processing large 3D volumes. In particular, the “zoom” function of the multi-dimensional image processing package (scipy.ndimage) of the SciPy ecosystem^2^ was used to resample each image by a factor of 0.5 using spline interpolation, resulting in images of shape 91 × 109 × 91 with isotropic 2 mm spatial resolution.

#### Body Mass Index

Data on weight were collected using a Tanita BC418MA body composition analyzer (Tanita Corporation of America, Inc., Arlington Heights, IL, United States). A Seca 240 cm height measure (Seca Deutschland, Hamburg, Germany) was used to obtain standing height measurement from participants. Body mass index was calculated as follows:

$$\mathrm{BMI}=\mathrm{weight}\mathrm{in}\mathrm{kilograms}/\mathrm{height}\mathrm{in}\mathrm{meters}{}^{2}$$

Further details on the anthropometric measurements can be obtained from the following URL: http://biobank.ndph.ox.ac.uk/showcase/refer.cgi?id=146620.

#### Age and Sex

The age of each participant was derived from the date of birth (data-fields 34, 52) and the date of the imaging visit (data-field 21,003 instance 2) and was given in years with precision to the month. Sex was self-reported (data-field 31) and coded as 0 for female and 1 for male.

### Prediction of Body Mass Index

#### Neural Network Architecture

We used a CNN to predict BMI. The prediction of the model is based on three inputs from each subject:

1. T1-weighted brain image in MNI152 space, encoded in a Numpy^3^ array of shape 91 × 109 × 91.
2. Chronological age of the participant in years with precision to the month.
3. Sex of the participant (0 for female and or 1 for male).

The output of the network is a single scalar corresponding to the predicted BMI of the subject.

**FIGURE 1:**
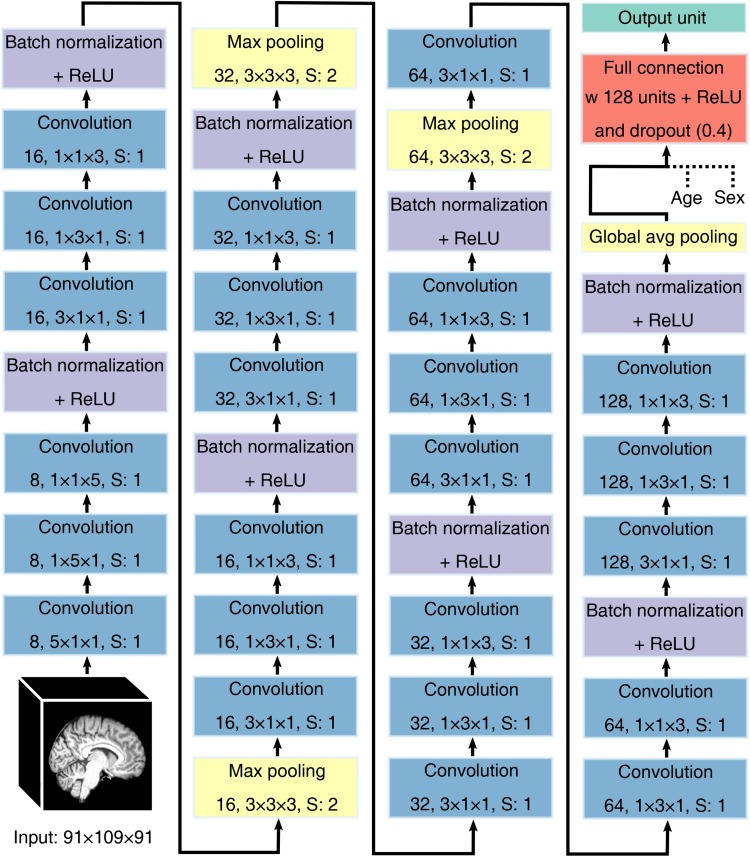
Schematic illustration of the architecture of the convolutional neural network used for predicting body mass index. The network comprises repeated blocks of 3D spatially separable convolutional layers followed by batch normalization and ReLU, with every other block followed by a pooling layer to subsample the input. Global average pooling is used to map the feature maps of the last block to a vector (with a single scalar for each feature map) that is fed into a fully connected hidden layer followed by a single output unit for BMI prediction. Dashed lines denote concatenation, S denotes stride.

A schematic illustration of the network architecture is given in Figure 1. The network comprises repeated blocks of 3D spatially separable convolutional layers followed by batch normalization (Ioffe and Szegedy, 2015) and rectified linear unit (ReLU) activation function (Nair and Hinton, 2010). In 3D spatially separable convolutional layers, instead of convolving the input with filters of shape *N* × *N* × *N*, a cascade of three asymmetric filters of shapes *N* × 1 × 1, 1 × *N* × 1, and 1 × 1 × *N* is used. Such a factorization of convolution operations reduces the computational cost by reducing the number of parameters (Szegedy et al., 2016) and has been used effectively in 3D medical image processing (Silva et al., 2018). Filter size is *N* = 5 (with a stride of 1) for the first set of convolution operations and *N* = 3 afterward. The number of filters is eight in the first convolutional layer and is doubled at regular intervals to enable the learning of a rich set of feature representations of the input brain image. All convolutional layers used SAME padding.

Every other batch normalization layer is followed by max pooling (filter shape 3 × 3 × 3, stride = 2) to subsample the input images, and global average pooling is implemented after the last batch normalization layer to calculate the average intensity value of each feature map computed by the last convolutional layer. The output of this operation, along with the values representing age and sex, is fed into a fully connected hidden layer with 128 units and ReLU activation function. This hidden layer is connected to a single output unit, the activation of which corresponds to the predicted BMI value.

The CNN has 231,681 parameters overall, out of which 230,961 parameters are trainable. The model was implemented in Python using TensorFlow 1.13.^4^ and the source code of the model along with the learnt parameters is available on GitHub: https://github.com/vaklip/cnn_3d_regression.

To examine whether information about age and sex was crucial for BMI prediction we also trained a network that was identical to the one described above, except that the values representing age and sex were not concatenated to the output of the global average pooling operation nor were they fed to the network in any other way.

#### Model Training

The weights of the convolutional and fully connected layers were initialized using Xavier initialization (Glorot and Bengio, 2010). The shifting and scaling parameters of the batch normalization layers were initialized to zeros and ones, respectively. The bias terms of the fully connected layers were initialized to 0.01. To train the network, we used mean squared error as the loss function, Adam optimizer (Kingma and Ba, 2014) with a learning rate of 0.0005 (momentum decay hyperparameter β1 = 0.9, scaling decay hyperparameter β2 = 0.999) and a batch size of eight. Dropout regularization (Wager et al., 2013; Srivastava et al., 2014) with a dropout rate of 0.4 was applied to the fully connected hidden layer during training.

The brain images of all participants were randomly assigned to disjoint training (*N* = 13938), validation (*N* = 2000), and test (*N* = 2000) sets. Only data in the training and validation sets were used for training and hyperparameter selection. The model was trained on the training set for a total of 50 epochs, and its performance was evaluated on the validation set after each epoch. A snapshot of the model parameters leading to the best validation set performance was restored and the final model was evaluated on the test set. Model performance is characterized by the MAE, standard deviation of the absolute error (STDAE), coefficient of determination (*R*^2^), root mean square error (RMSE), and Pearson’s correlation coefficient (*r*) between the true and predicted BMI values.

A single NVIDIA Quadro M4000 GPU was used to train the CNN, with a runtime of about 1 h per epoch.

#### Transfer Learning

We used transfer learning to investigate the generalizability of our approach. Transfer learning refers to the method of training a neural network on one dataset (the source domain) and then adapting the model to a different dataset and/or task (the target domain) by transfer and fine-tuning of the previously learned model weights. In our case, the UK Biobank dataset constituted the source domain and the Information eXtraction from Images (IXI) dataset^5^ including brain MR images from multiple sites in London constituted the target domain. We included the T1-weighted MR images of 269 subjects from the IXI dataset who fell into the age range corresponding to the UK Biobank sample: 177 females aged between 44 and 78 years (mean ± SD = 60.50 ± 8.32 years) and 115 males aged between 44 and 79 years (mean ± SD = 59.48 ± 9.05 years). These images were recorded using Philips Intera 3T (*N* = 96; Hammersmith Hospital) and Philips Gyroscan Intera 1.5T (*N* = 173; Guy’s Hospital) scanners and a FOV of 150 × 256 × 256 and spatial resolution of 1.2 mm × 0.938 mm × 0.938 mm. Images recorded at a third location (Institute of Psychiatry using a GE 1.5T system) were omitted from the current analysis due to the very low number of participants that matched the given age range (*N* = 23). In-house image preprocessing was limited to spatial normalization to MNI152 space and skull-stripping using the SPM12 toolbox^6^ and custom-made scripts running on MATLAB 2015a (MathWorks Inc., Natick, MA, United States).

Images were randomly divided into disjoint training (*N* = 197), validation (*N* = 36), and test sets (*N* = 36). The weights of the network were initialized to those learnt on the UK Biobank dataset and then trained on the IXI dataset for 50 epochs, using data augmentation (random rotations of maximum 5 degrees and translations of 10 voxels). The neural network architecture and training hyperparameters were the same as those used for training on UK Biobank data. A snapshot of the model parameters leading to the best validation set performance (evaluated at the end of each epoch) was restored and the final model was evaluated on the test set.

### Localizing Brain Regions Relevant for BMI Prediction

In order to obtain localization maps highlighting brain regions that are important for BMI prediction, we used a modified version of the Grad-CAM (Selvaraju et al., 2017). The Grad-CAM method aims to provide visual explanations for the decisions made by a wide variety of CNNs. It uses the gradients of a given target concept flowing into the final convolutional layer to produce a coarse localization map that highlights regions in the input image that are important for predicting that concept. We applied two modifications to the original method. First, we adapted it for processing 3D images, similarly to (Wang et al., 2019). We computed the gradient of the predicted BMI-score *y* with respect to the feature maps *A*^*n*^ of the last convolutional layer, and performed global average pooling on these gradients to obtain an importance weight α_*n*_ for each feature map:

(1) $$\alpha_{n}=\frac{1}{Z}\,\sum_{i}\sum_{j}\sum_{k}\frac{\partial y}{\partial A_{i\,j\,k}^{n}}$$

where *Z* is the number of units in a feature map. Then, the weighted combination of the features maps was calculated to obtain the localization map *L* ∈ *ℝ*^*u*×*v*×*w*^:

(2) $$L=\sum_{n}\alpha_{n}\,A^{n}$$

In the original formulation of Grad-CAM, which was developed to provide class-discriminative visualizations, a ReLU was applied to *L* in order to highlight features that have a positive influence on the class of interest, as negative values would likely belong to other classes (Selvaraju et al., 2017). Here, since our CNN performed a regression task with a single output unit, and hence we were interested in features that have either positive or negative influence on predicted BMI, we omitted this step.

Localization maps were computed for each individual in the UK Biobank test set. They were upsampled to match the size of the input images using spline interpolation (for details, see section “Neuroimaging”). Intensity values were standardized to have zero mean and unit variance. As all brain images were registered to MNI152 space, a voxelwise grand average localization map across all test subjects could be computed. The resulting map was thresholded at two standard deviations from the mean and superimposed on the ch2bet MRIcron^7^ template to visualize regions in the brain that made a strong contribution to BMI prediction. To investigate the robustness of the results, a grand average localization map was also computed for the training set. This localization map was visually indistinguishable from the one obtained for the test set.

### Examining the Relationship Between BMI and Brain Volumetric and Morphometric Variability

Based on the visualization provided by the modified Grad-CAM method, we performed further exploratory analyses to investigate the association between BMI and morphological variability in the human brain using the UK Biobank data. To this end, we randomly selected a subset of 200 participants from the test set, with the only constraint being that the male–female ratio and the distribution of chronological age and BMI remain similar to those in the overall test set. We used FreeSurfer 6.0^8^ to automatically parcellate the cortical surface and segment the subcortical structures in the anatomical images of these subjects (Dale et al., 1999; Fischl et al., 1999). Then we investigated the relationship between different measures of cortical and subcortical anatomy—estimated by FreeSurfer—and the true BMI of participants, as detailed below.

#### Subcortical Segmentation

The volume-based stream of FreeSurfer (Fischl et al., 2002, 2004) was used to quantify the volumes of left and right hemisphere subcortical structures. Subcortical structures were selected for volumetric analysis based on the regions highlighted in the localization map produced by the modified Grad-CAM method. We computed partial correlations to examine the relationship between subcortical structure volume and BMI while controlling for chronological age, sex, and overall subcortical gray matter volume. We controlled for the former two variables since they were added as covariates to the CNN model which was therefore able to adjust for structural differences between individuals of different age and sex. Partial correlations were calculated using Statistica 13.4. (TIBCO Software Inc., Palo Alto, CA, United States).

#### Cortical Parcellation

The surface-based stream of FreeSurfer (Dale et al., 1999; Fischl et al., 1999) was used to construct models of the boundaries between white matter and cortical gray matter (the white surface), and between gray matter and the cerebrospinal fluid (the pial surface). The triangular tessellation of these surfaces allows for the calculation of several morphometric measures at each location (vertex) of the cortex, including cortical thickness, area, and curvature. We investigated the relationship between these three measures and BMI using FreeSurfer’s Query, Design, Estimate, Contrast (QDEC) tool. Specifically, after smoothing individual subject data to the average surface with a 10-mm full-width at half maximum Gaussian kernel, a general linear model (GLM) with one of the morphometric measures as dependent variable was applied at each vertex, accounting for the effects of age, sex, and total cortical gray matter volume. False discovery rate (FDR) correction (threshold at 0.05) was applied to reduce Type I. errors associated with multiple comparisons.

Based on the grand average localization map, we directly investigated the association between the morphology of the right middle temporal gyrus and BMI. In particular, we computed partial correlations to examine the relationship between BMI and surface area, mean thickness and curvature while controlling for age, sex, and total cortical gray matter volume.

## Results

### BMI Prediction

**FIGURE 2:**
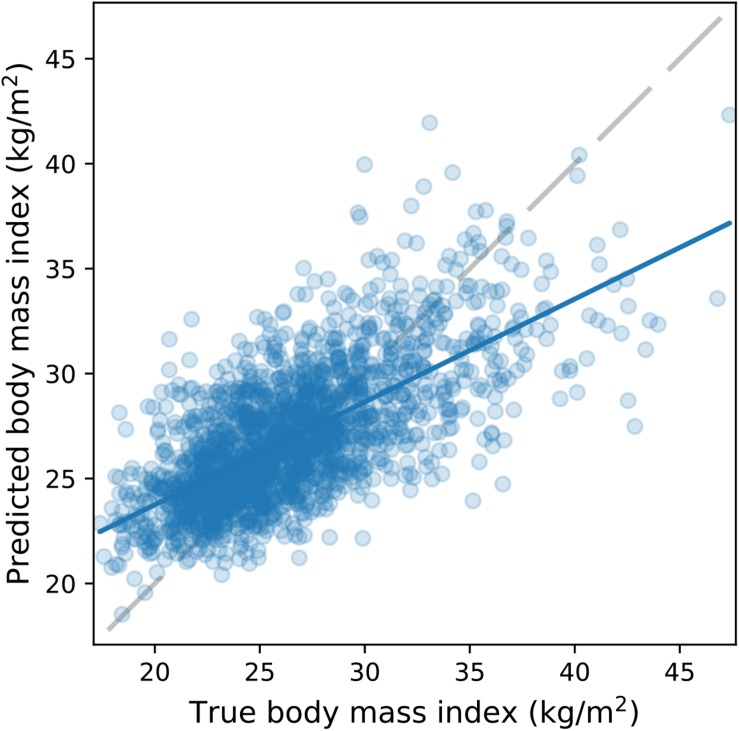
BMI prediction accuracy on the UK Biobank dataset. The scatterplot depicts the true (horizontal axis) and the CNN-predicted BMI (vertical axis) on the test set (*N* = 2000). A least squares regression line (continuous blue) is superimposed on the scatterplot.

Overall, results showed that our CNN model can be used to predict BMI with high accuracy. Prediction error on the validation set reached a minimum after 32 epochs (MAE = 2.41 kg/m^2^, STDAE = 1.93 kg/m^2^). The model generalized well to the brain images in the test set (Figure 2): MAE = 2.48 kg/m^2^; STDAE = 2.09 kg/m^2^; RMSE = 3.24 kg/m^2^; Pearson *r* = 0.68; *R*^2^ = 0.44.

When training the network without feeding information about age and sex to it, it took longer to reach a minimum of prediction error on the validation set (after 41 epochs, MAE = 2.36 kg/m^2^, STDAE = 2.09 kg/m^2^). Nevertheless, the model generalized well to the test set images: MAE = 2.41 kg/m^2^; STDAE = 2.11 kg/m^2^; RMSE = 3.20 kg/m^2^; Pearson *r* = 0.7; *R*^2^ = 0.46.

**FIGURE 3:**
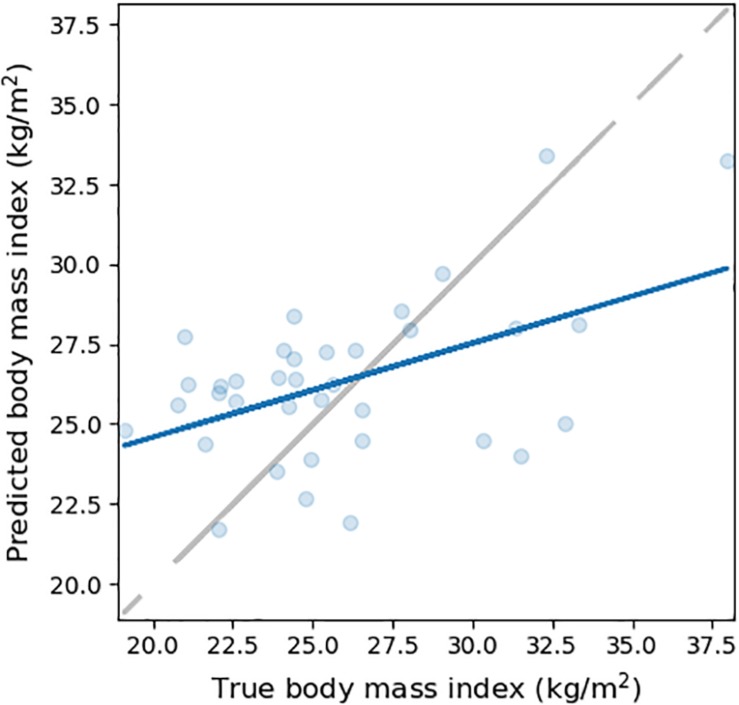
BMI prediction accuracy on the IXI dataset. The scatterplot depicts the true (horizontal axis) and the CNN-predicted BMI (vertical axis) on the test set (*N* = 36). A least squares regression line (continuous blue) is superimposed on the scatterplot.

When fine-tuning learned weights on the IXI dataset, validation error reached a minimum after 44 epochs (MAE = 2.53 kg/m^2^; STDAE = 2.00 kg/m^2^). We obtained reasonable BMI prediction on the IXI test set (Figure 3; MAE = 3.00 kg/m^2^; STDAE = 2.12 kg/m^2^; RMSE = 3.67 kg/m^2^; Pearson *r* = 0.49; *R*^2^ = 0.21), albeit it was below the performance obtained in the case of the UK Biobank dataset.

### Localization Map

**FIGURE 4:**
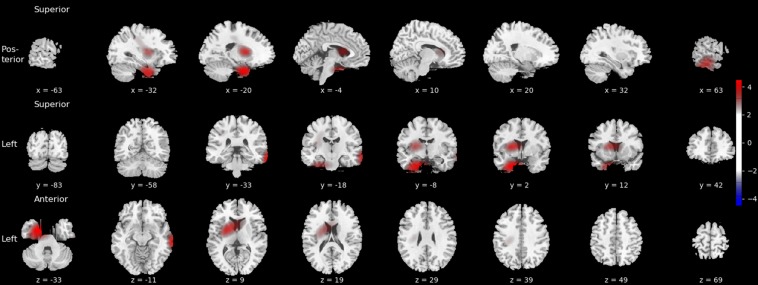
Grand average localization map highlighting brain regions that strongly contribute to predicted BMI. Activation values are *z*-scored and thresholded at | *Z*| > 2. The localization map is superimposed on the ch2bet MRIcron template with MNI coordinates displayed below each slice.

The grand average localization map across all the 2000 subjects’ images in the test set is depicted in Figure 4. The map highlights several regions that, on average, have a strong influence on predicted BMI. These regions include the left caudate, the left medial temporal lobe in the vicinity of the amygdala, and the lateral surface of the right temporal cortex, encompassing the middle temporal gyrus.

### Brain Volumetric and Morphometric Analyses

**FIGURE 5:**
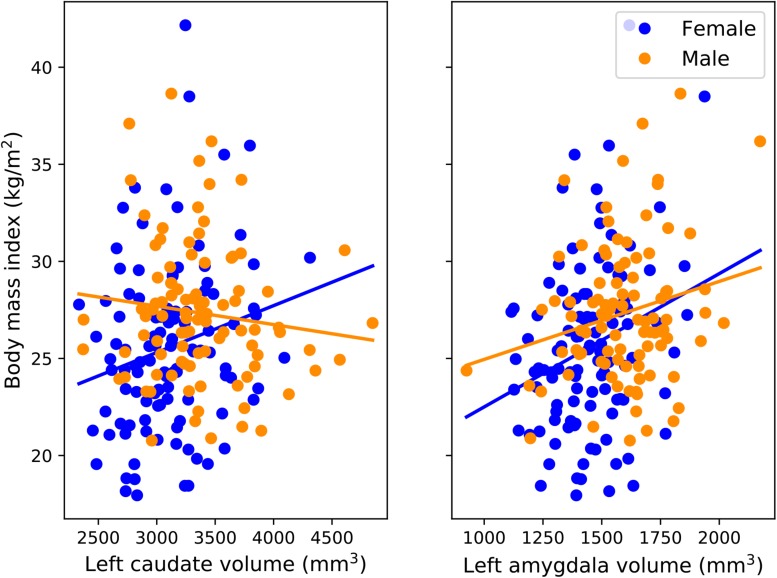
BMI and subcortical volumes. Scatterplots depict the volumes of the caudate **(left panel)** and amygdala **(right panel)** in the left hemisphere and the true BMI values of male (*N* = 93) and female (*N* = 107) subjects in the test set.

Based on the localization map, two subcortical regions, the left caudate and amygdala, were selected for volumetric analysis in a subset of the test subjects (Figure 5). On the one hand, there was no significant partial correlation between the volume of the caudate and the true BMI of the subjects when controlling for chronological age, sex, and overall subcortical gray matter volume (*r* = 0.028, *p* = 0.7). This may be accounted for by sex differences in the relationship between caudate volume and BMI (Figure 5, left panel). On the other hand, a significant partial correlation between the volume of the amygdala and BMI was observed (*r* = 0.19, *p* = 0.008), showing that increased BMI is associated with increased amygdalar volume.

**FIGURE 6:**
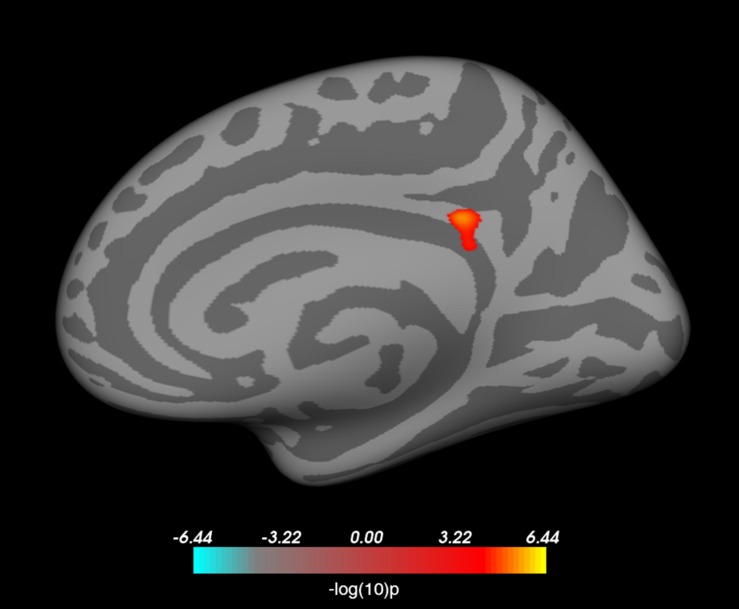
Vertex-wise analysis of surface area using FreeSurfer. BMI is significantly associated with surface area in a right hemisphere cluster encompassing the isthmus cingulate cortex (when age, sex, and total cortical gray matter volume are controlled for). The cluster survived false discovery rate correction at threshold *p* < 0.05.

Regarding the analysis of cortical morphometry, no significant association between BMI and cortical thickness or curvature was observed after correcting for multiple comparisons (FDR threshold at 0.05). However, a positive relationship was observed between BMI and the area of the isthmus cingulate in the right hemisphere (Figure 6). The direct tests (partial correlations) of the association between BMI and morphological measures of the right middle temporal gyrus yielded no significant results.

## Discussion

In this proof-of-concept study, we established that a deep CNN can be used to predict individual BMI with high accuracy, based on a single structural MRI brain scan and information about age and sex. This finding is in line with the results of several previous studies showing gray and white matter structural alterations in obese individuals (Brooks et al., 2013; Kullmann et al., 2015; Dekkers et al., 2019). We also demonstrated that gradient-based visualization can be used effectively to highlight brain regions that play an important role in BMI prediction. More specifically, we used the Grad-CAM method, based on the gradient information flowing into the last convolutional layer of the CNN (Selvaraju et al., 2017), and adapted it to the context of regression using 3D images to identify brain regions that, on average, made a strong contribution to predicted BMI values. Our results suggest that, in addition to conventional neuroimaging methods and analytical techniques, the use of DL along with visual explanations for model predictions is a suitable approach for identifying the brain structural correlates of individual variability in body weight.

In particular, the localization map produced by the Grad-CAM method highlighted a set of brain regions including a portion of the left medial temporal lobe in the vicinity of the amygdala. The relationship between amygdalar volume and BMI was also confirmed by using FreeSurfer-based subcortical segmentation and partial correlation correcting for age and sex, which showed that higher BMI was associated with larger amygdalar volume. Previous studies using voxel-based (Taki et al., 2008) and tensor-based morphometry (Raji et al., 2010) found a relationship between BMI and the volume of gray and white matter in the medial temporal lobe. With regard to the amygdala, a positive relationship between BMI and amygdalar volume was already found in children and adolescents (Perlaki et al., 2018), young adults (Orsi et al., 2011), and elderly subjects (Widya et al., 2011); although a negative association has also been described (Kharabian Masouleh et al., 2016). Taken together, these results show that the DL approach paired with gradient-based visualization and more conventional neuroimaging methods provide converging evidence regarding the link between body weight and amygdalar structure. This is in accordance with the results of functional neuroimaging studies providing evidence for the involvement of the amygdala in processing visual food cues (van der Laan et al., 2011; Tang et al., 2012; van Bloemendaal et al., 2014).

Besides the commonalities, several discrepancies have been observed between the results of the Grad-CAM-based localization and the vertex-wise analysis using FreeSurfer. On the one hand, the vertex-wise analysis yielded a significant association between BMI and the surface area in a region corresponding to the isthmus cingulate in the right hemisphere. While at least one previous study reported a relationship between BMI and the morphology of the posterior cingulate cortex (Kharabian Masouleh et al., 2016), this region did not light up in the Grad-CAM-based localization map. On the other hand, several other brain structures were deemed important based on the localization map, in the case of which the conventional automatic brain segmentation approach failed to confirm an association with BMI, namely the lateral surface of the right temporal cortex and a region encompassing the left caudate nucleus. With regard to the latter, a previous study has shown that the volume of the caudate heads bilaterally show a positive association with BMI in men, after adjusting for age, lifetime alcohol intake, history of hypertension, and diabetes mellitus (Taki et al., 2008). Sex differences have also been shown to be manifest regarding the relationship between total body fat and caudate volume (Dekkers et al., 2019). Our results regarding the association with BMI are also indicative of such differences (Figure 5, left panel). In addition, the discrepancy between our observations with DL and conventional approaches is likely to stem from the differences in the applied methodologies as well. In our study, we used FreeSurfer for the automated segmentation of predefined subcortical structures and examined the linear relationship between BMI and a single scalar estimate of the volume of the caudate. FreeSurfer segmentation includes a series of pre-processing steps applied to the MRI volumes, followed by labeling the volumes based on a probabilistic atlas built from a set of hand-labeled images, as well as subject-specific measurements (Fischl et al., 2002, 2004). In contrast, the CNN is fed with minimally preprocessed images and learns a series of transformations to map those images to the corresponding BMI values. Each of these transformations map the representation of the input at one level into a representation at a slightly more abstract level (LeCun et al., 2015). Compared to the conventional automated brain segmentation methods, visualizations based on these more abstract representations may provide additional information with regard to the relationship between brain architecture and body weight. Similarly, several recent studies applied the Grad-CAM method to highlight brain regions that made an important contribution to predicting depression and epilepsy (Pominova et al., 2018), brain age (Bermudez et al., 2019), and Alzheimer’s disease (Feng et al., 2018) based on structural MRI data.

Besides being a promising tool for neuroscientific investigation, brain-predicted BMI may also have practical utility. We managed to adapt the CNN model to a novel dataset, suggesting that our method is more generally applicable to a variety of different MR scanner types. Coming back to the relationship between the amygdala and body weight, this brain structure has been shown to be involved in the evaluation of food cues (Siep et al., 2009) and to constitute a part of a neural circuitry involved in the regulation of food craving (Dietrich et al., 2016). In a recent review, it has been argued that structures of the medial temporal lobe, in particular the amygdala and the hippocampus, may play an important role in the regulation of body weight, and that the amygdala is crucial for the regulation of feeding behavior based on environmental cues (Coppin, 2016). Based on the localization map produced by the Grad-CAM method, it is reasonable to hypothesize that brain-predicted BMI may be related to individual differences in the processing of food stimuli and cue-induced feeding. On this basis, one intriguing possibility is that increased brain-predicted BMI relative to the actual BMI might reflect a greater propensity to weight gain. This mode of application is similar to how the difference between brain-predicted and chronological age might have clinical utility (Cole and Franke, 2017). However, it is important to note that brain structural alterations might not be the cause but the consequence of obesity. In fact, obesity-driven neuroinflammation has been shown to affect several brain regions including the hippocampus and the amygdala (Guillemot-Legris and Muccioli, 2017). Further research is necessary to examine whether and how brain-predicted BMI is related to pathophysiological processes and eating behavior.

## Conclusion

Our findings provide proof of concept that individual BMI can be predicted with high accuracy from a single MRI scan using DL methods and suggest a relationship between the morphology of subcortical structures and body weight.

## Data Availability Statement

Publicly available datasets were analyzed in this study. This data can be found here: this research has been conducted using the UK Biobank Resource under Application Number 27236. All analyses reported in this paper include participants from the UK Biobank population cohort (https://www.ukbiobank.ac.uk/). The source code of the presented model along with the learnt parameters is available on GitHub: https://github.com/vaklip/cnn_3d_regression.

## Ethics Statement

The studies involving human participants were reviewed and approved by UK Biobank Research Ethics Committee (REC; approval number: 11/NW/0382). The participants provided their written informed consent to participate in this study.

## Author Contributions

All authors listed have made a substantial, direct and intellectual contribution to the work, and approved it for publication.

## Conflict of Interest

The authors declare that the research was conducted in the absence of any commercial or financial relationships that could be construed as a potential conflict of interest.

## Footnotes

1 https://www.ukbiobank.ac.uk/

2 https://scipy.org/

3 https://numpy.org/

4 https://www.tensorflow.org

5 https://brain-development.org/ixi-dataset/

6 http://www.fil.ion.ucl.ac.uk/spm/software/spm12/

7 https://people.cas.sc.edu/rorden/mricron/index.html

8 http://surfer.nmr.mgh.harvard.edu
